# Exploring the Electronic Interactions of Adenine, Cytosine, and Guanine with Graphene: A DFT Study

**DOI:** 10.1002/open.202400350

**Published:** 2024-11-19

**Authors:** Jawaher Qasem, Baliram Lone

**Affiliations:** ^1^ Nanomaterials Research Laboratory Department of Physics Vinayakrao Patil Mahavidyalaya Vaijapur, Dist. Sambhajinagar Maharashtra 423701 India; ^2^ Department of Physics Taiz University Taiz 380015 Yemen

**Keywords:** Density functional theory, Pure graphene, Adenine, Cytosine, Guanine, HOMO-LUMO

## Abstract

This study has provided new insights into the interaction between graphene and DNA nucleobases (adenine, cytosine, and guanine). It compares how each nucleobase interacts with graphene, examining their selectivity and binding energy. The research also explores how these interactions impact the electronic properties of graphene, showing potential applications in graphene‐based biosensors and DNA sequencing technologies. Additionally, the findings suggest potential uses in DNA sensing and the functionalization of graphene for various biomedical applications. This study employs density functional theory (DFT) methods, utilizing the B3LYP functional with the 6‐311G basis set, to explore the electronic interactions between DNA nucleobases (adenine, cytosine, and guanine) with pure graphene (Gr). We investigate various properties, including adsorption energy, HOMO‐LUMO energy levels, charge transfer mechanisms, dipole moments, energy gaps, and density of states (DOS). Our findings indicate that cytosine interacts most favorably with graphene through its oxygen site (Gr‐Cyt‐O), exhibiting the strongest adsorption. Additionally, adenine's interaction significantly enhances its electronegativity and chemical potential, particularly at the nitrogen position, while decreasing its electrophilicity. Guanine, characterized by the smallest energy gap, demonstrates the highest conductivity among the nucleobases. These results suggest that graphene possesses advantageous properties as an adsorbent for guanine, highlighting its potential applications in biosensor technology.

## Graphical abstract

Density Functional Theory (DFT) explores Pure Graphene's ability to detect Adenine, Cytosine, and Guanine by analyzing adsorption energy, HOMO‐LUMO levels, gap, dipole moment, charge transfer, and density of states. Results show Guanine reduces the HOMO‐LUMO gap, enhancing Graphene's conductivity, making the Graphene‐Guanine complex a promising candidate for advanced Guanine‐based biosensors.

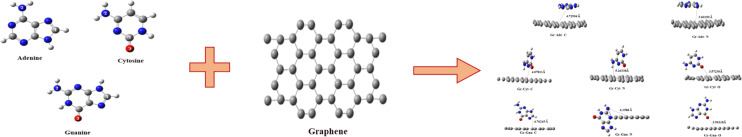

## 1 Introduction

The DNA molecule, characterized by its double helix structure, is composed of two intertwined strands connected by hydrogen bonds between complementary nucleobases. In this structure, adenine (A) pairs with thymine (T), and cytosine (C) pairs with guanine (G), establishing the fundamental units of genetic information. These nucleobases are vital for the genetic code, responsible for storing and transmitting genetic data in all living organisms. Adenine, cytosine, and guanine are integral to various biological processes, such as DNA replication, repair, transcription, and translation. The specific sequences of these nucleobases encode the instructions for synthesizing proteins, which carry out vital functions within cells. Understanding the interactions of these nucleobases is crucial for advancements in genetic research and biotechnology. Recent studies have emphasized the importance of these nucleobases in maintaining genetic accuracy and the potential for their interchangeability in nucleic acid structures. The unique properties of these nucleobases have led to their widespread use in various biological processes, including energy metabolism and cell signaling. The interactions between nucleobases are crucial to the genetic code and are integral to various biological processes. Grasping these interactions is vital for progress in genetic research and biotechnology.[^1^, ^2^, ^3^, ^4^, ^5^] Graphene is composed of a monolayer of carbon atoms arranged in a two‐dimensional hexagonal lattice, has garnered much interest due to its extraordinary characteristics. Graphene's extraordinary properties make it a versatile material for a wide range of applications. Its exceptional electrical conductivity, large surface area, and strength make it an ideal material for energy storage, sensors, water purification, composite materials, solar cells, and conductive inks and coatings.

Mechanical strength and lightweight nature of graphene are particularly advantageous for developing strong, lightweight composite materials. In biotechnology, graphene's biocompatibility and ability to facilitate cellular interactions have enabled advancements in innovative biosensors and drug delivery systems. These unique properties drive graphene's extensive research and application in diverse technological fields. Recent studies have focused on synthesizing and characterizing graphene and its integration into various devices and systems. Graphene's exceptional characteristics have catalyzed its use in energy storage, sensors, and biomedical applications, highlighting its significant potential in electronics, biotechnology, and beyond.[^6^, ^7^, ^8^] Investigating nucleobase‐graphene interactions is vital for developing cutting‐edge biosensors, DNA sequencing technologies, and nanodevices. Recent studies have demonstrated the effectiveness of graphene‐based materials in detecting DNA, RNA, small molecules, and proteins due to their exceptional sensitivity and selectivity. Studies have demonstrated that van der Waals forces are crucial in the interaction between nucleobases and graphene. These interactions significantly improve the effectiveness of biosensors and support DNA sequencing techniques.Despite these advances, considerable gaps remain in the theoretical understanding of these interactions, necessitating further investigation to fully optimize graphene‐based applications. Recent research has also explored the deposition of DNA nanostructures on graphene, enabling precise molecular organization at the nanoscale and paving the way for innovative biosensors and sequencing technologies. Thus, a comprehensive study of nucleobase‐graphene interactions is crucial, combining both theoretical and experimental approaches to fully realize the potential of graphene in biomedical applications.[^9^, ^10^, ^11^, ^12^] Density Functional Theory (DFT) is a prevalent computational quantum mechanical approach, crucial for investigating the electronic structures of atoms, molecules, and condensed matter systems. DFT simplifies the complex many‐body problem of interacting electrons in a system by utilizing electron density instead of wave functions, making it computationally efficient and accurate for various applications. Advantage of DFT is its ability to provide detailed insights into molecular interactions, which is crucial for understanding binding mechanisms and adsorption properties at the atomic level. Additionally, DFT excels in predicting electronic properties such as band structure and density of states, which are vital for designing materials with specific electronic characteristics.[^13^, ^14^, ^15^]

Recent advancements in DFT have improved its accuracy and applicability, making it an invaluable tool in materials science and nanotechnology research. DFT's computational efficiency and versatility have revolutionized the study of electronic structures, enabling researchers to explore a wide range of materials and systems with precision and efficiency.[^16^, ^17^] The study of how nucleobases interact with graphene is essential for developing advanced biosensors and nanodevices. Graphene has unique properties such as high electrical conductivity and a large surface area, making it a valuable material for these applications. Density Functional Theory (DFT) plays a crucial role in these studies by accurately predicting binding energies, electronic structures, and interaction mechanisms.[^18^, ^19^] Recent DFT studies have provided detailed insights into the adsorption behavior of adenine, cytosine, and guanine on graphene. These studies have highlighted the importance of π‐π stacking interactions and charge transfer phenomena. Computational predictions like these are crucial for designing graphene‐based materials with optimized performance for DNA sequencing and molecular sensing applications.^[20]^ The precision of DFT in modeling these complex interactions enables researchers to explore new functionalization strategies and tailor graphene's properties for specific biotechnological uses. Graphene‐based biosensors have demonstrated remarkable performance, exhibiting exceptional sensitivity, selectivity, stability, and a broad detection range.[^21^, ^22^] Investigating the interactions between nucleobases and graphene using density functional theory (DFT) is vital for the development of cutting‐edge biosensors and nanodevices. The knowledge acquired from these analyses is pivotal in crafting graphene‐based materials with enhanced performance for applications in DNA sequencing and molecular sensing.^[23]^ This study aims to investigate the interactions of adenine, cytosine, and guanine with pure graphene using Density Functional Theory (DFT). Through DFT, we seek to gain a detailed understanding of the binding mechanisms of these fundamental DNA nucleobases on graphene surfaces. Our focus will be on identifying the optimal binding configurations of adenine, cytosine, and guanine on graphene, determining the most stable adsorption geometries. We will also calculate the adsorption energies for each nucleobase to quantify the interaction strength with graphene. Additionally, we will explore the electronic properties of the nucleobase‐graphene complexes by analyzing changes in electronic structure, such as charge transfer and density of states, induced by nucleobase adsorption on graphene. The insights from this study are expected to deepen our understanding of nucleobase‐graphene interactions, providing valuable information for designing and developing graphene‐based biosensors, nanodevices, and other biotechnological applications.

## 2 Methodology

Gauss View 5.0 was utilised in order to construct the molecular models of the pure graphene sheet (Gr) as well as the DNA nucleobases adenine (Ade), cytosine (Cyt), and guanine (Gua).[^24^, ^25^] Figure 2 shows the result of our efforts to create a 4×4 pure graphene (Gr) sheet with 42 carbon atoms. This model was based on previously published graphene structures.^[26]^ DFT‐based methods, specifically B3LYP with the 6‐311G basis set,^[27]^ interactions of graphene (Gr) adsorbed by the DNA nucleobases adenine, cytosine, and guanine were investigated. The study assessed electronic properties such as Natural Bond Orbital (NBO) analysis, HOMO‐LUMO gaps, and Density of States (DOS) of the complex systems, utilizing the Gaussian09 software package.^[28]^ For isolated and complex systems, Gr was defined at a 1×1×3 Monkhorst‐Pack grid for k‐point sampling of the Brillouin zone, adjusted to 3×3×1 for integration. The optimisation process was carried out until the residual forces reached a normalised value of 0.01 eV/Å.[^28^, ^29^]

The adsorption energy E_ads_ of the complex system is calculated by Eq. 1:

(1) Eads=Ecomplex-Esurface-Eadsorbate

The variables E_complex_, E_surface_, and E_adsorbate_ represent the total energy of the adsorbate‐surface complex, the clean surface, and the isolated adsorbate molecule, respectively. The HOMO and LUMO correspond to the energy levels with the highest and lowest electron occupancy, respectively. The energy gap, or bandgap, is the energy difference between the HOMO and LUMO and is crucial for determining the optical and electronic properties of a material. The energy gap is calculated using the Eq. 2:

(2) Egap=ELUMO-EHOMO......

The energy of the LUMO is referred to as E_LUMO_, while the energy of the HOMO is referred to as E_HOMO_.

Global Reactivity Descriptors (GRDs) offer insights into the general reactivity characteristics of a molecule. The characteristics, such as chemical hardness η, global electrophilicity ω, chemical potential μ, and chemical softness S, are obtained from electronic structure and computed using the B3LYP/6‐31G method in the following Eq. (3)–Eq. 6:

(3) ω=μ2/2η......

(4) μ=[ELUMO+EHOMO]/2......

(5) η=[ELUMO-EHOMO]/2......

(6) S=1/η......

These descriptors were computed to analyze the reactivity and stability of the nucleobase‐graphene complexes.[^31^, ^32^, ^33^, ^34^]

## 3 Results and Discussion

### 3.1 Structural Analysis

**Figure 1:**
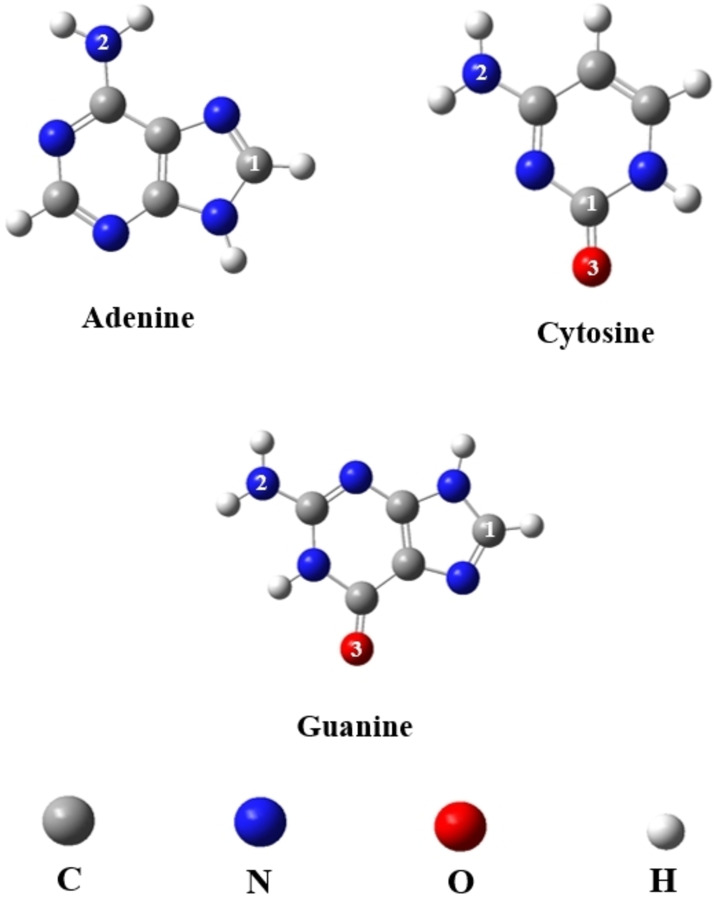
Schematic of DNA nucleobases adenine, cytosine, and guanine molecular structure.

We optimized molecular geometries of a complex system that includes a pure graphene (Gr) sheet interacting with the DNA nucleobases adenine (Ade), cytosine (Cyt), and guanine (Gua) through specific atoms. Adenine (Ade) interacts with two atoms (C, N), Cytosine (Cyt) and Guanine (Gua) interact with three atoms (C, N, O) during adsorption on the surface of Pure Graphene (Gr). Figure 1. shows the chemical structures and molecular configurations of these DNA nucleobases: adenine (Ade), cytosine (Cyt), and guanine (Gua). The color scheme used for the atoms is as follows: C (gray), N (blue), O (red), and H (white).

**Figure 2:**
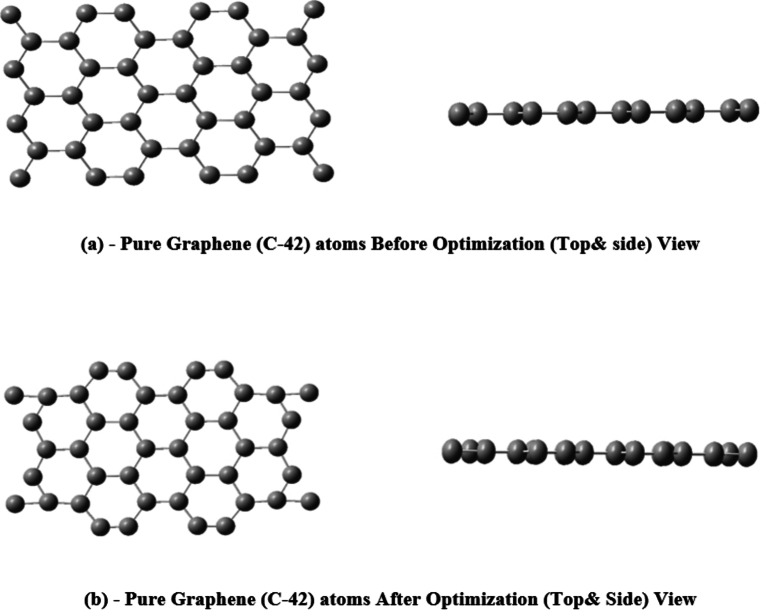
Schematic representation of the structure of pure Graphene (Gr): (a) Before optimization and (b) after optimization, showing both top and side views.

In pure graphene sheets (Gr), the bond lengths in pure graphene sheets (Gr) exhibit a range of values, specifically between 1.497 Å and 1.527 Å. During deformation, a particular transformation from sp2 to sp3 hybridization occurs at the locations where atoms are doped. Figure 2. depicts the top and side views of the pure graphene structure, showing its configuration before and after optimization.

### 3.2 Adsorption of DNA Nucleobases Adenine, Cytosine, and Guanine on Pure Graphene Surface

**Figure 3:**
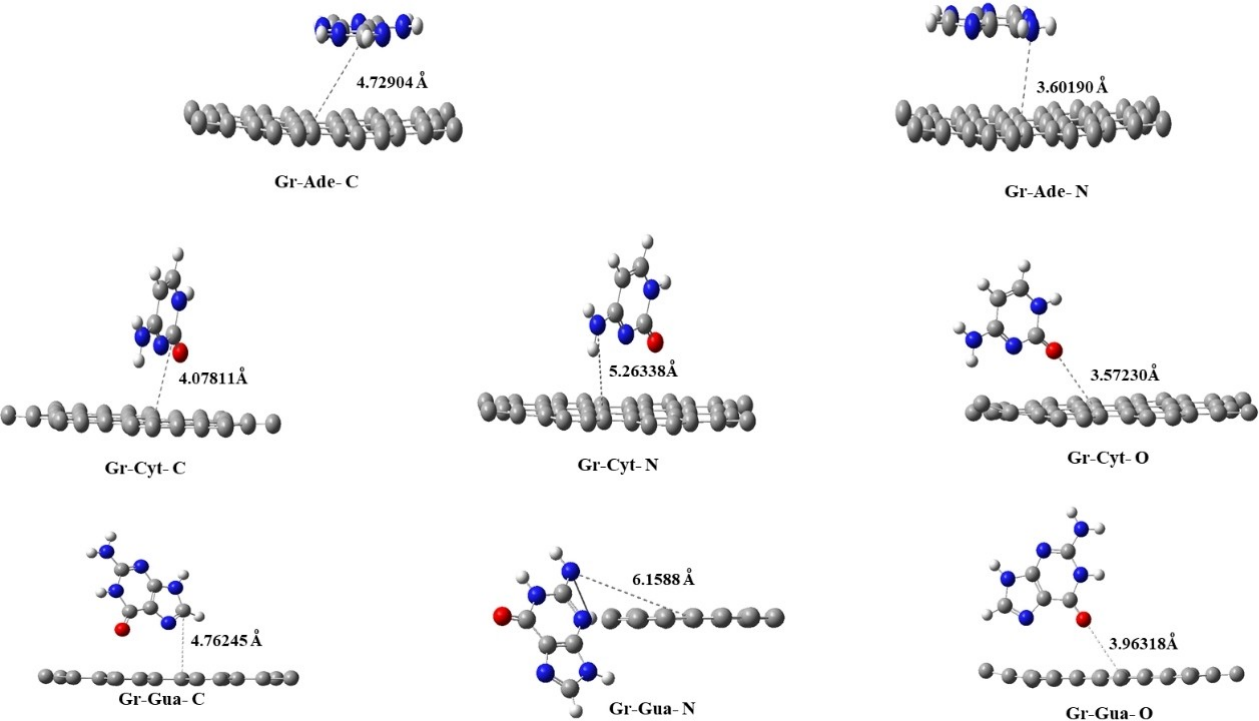
The fully optimized complex graphene (Gr) system with DNA nucleobases is adsorbed at various positions.

After optimizing the molecular geometries of the complex systems, we noticed that the bond lengths resulting from the interactions between the DNA nucleobases (adenine, cytosine, and guanine) on the pure graphene surface varied based on the potential interactions of the nucleobase atoms with the graphene surface. This is shown in Figure 3.

We investigated how DNA nucleobases interact with a graphene (Gr) surface, focusing on their active sites Figure 1. The adsorption mechanisms of each DNA nucleobase onto the graphene surface were studied, and the results are depicted in Figure 3. Our analysis, conducted using quantum chemical methods, revealed a favorable interaction between DNA nucleobases and pure graphene during adsorption, as shown in Figure 3. This conclusion is supported by the calculated adsorption energies and bond lengths detailed in Table 1 The potential adsorption sites on the DNA nucleobase molecules include −C, −N, and =O groups. The adsorption energy sign indicates the thermodynamic favorability of the adsorption process. A negative adsorption energy reflects an exothermic and thermodynamically favorable process. The more negative the value, the stronger the interaction between the adsorbate and the surface. On the other hand, a positive adsorption energy reflects an endothermic and thermodynamically unfavorable process, suggesting repulsion between the adsorbate and the surface and making adsorption unlikely. In summary, a negative adsorption energy indicates favorable adsorption, while a positive value suggests unfavorable adsorption. The magnitude of the adsorption energy reflects the strength of the interaction.[^35^, ^36^]

**Table 1:** The physical parameters include the adsorption energy (E_ads_), NBO charge transfer (Q_NBO_), binding distance (d), Mulliken charge transfer (Q_Mu_), and Dipole moment (μD)of the complex system, which involves the adsorption of DNA nucleobases onto the Gr sheet at various places.

Complex system	E_ads_ (eV)	d (A.U.)	Q_Mu_ (e)	Q_NBO_ (e)	μD (Debye)
Gr‐Ade‐C	26.495495	4.7290	0.0160	0.2460	2.0173
Gr‐Ade‐N	26.495169	3.6019	−0.7700	−0.6700	2.4145
Gr‐Cyt‐C	−0.406375	4.0781	0.4070	0.6780	8.6692
Gr‐Cyt‐N	−0.379164	5.2634	−0.7860	−0.7100	8.0182
Gr‐Cyt‐O	−0.588474	3.5723	−0.3310	−0.4810	8.8437
Gr‐Gua‐C	−0.406239	4.7625	0.0260	0.2140	9.2948
Gr‐Gua‐N	−0.119104	6.1588	−0.7440	−0.7090	4.2566
Gr‐Gua‐O	−0.447682	3.9632	−0.3480	−0.4860	9.2168

For various DNA nucleobase‐graphene interactions, the table analysis requires deciphering the adsorption energy (Eads), Mulliken charge transfer (QMu), binding distance (d), NBO charge transfer (QNBO), and Dipole moment (μD).Adsorption Energy (E_ads_).

- The most negative adsorption energy represents the most favorable interaction. (Gr‐Ade‐C) and (Gr‐Ade‐N) have positive adsorption energies (26.495495 eV and 26.495169 eV, respectively), indicating thermodynamically unfavorable interactions. On the other hand, (Gr‐Cyt‐C) has (−0.406375 eV), (Gr‐Cyt‐N) has (−0.379164 eV), and (Gr‐Cyt‐O) has (−0.588474 eV) with negative adsorption energies, suggesting favorable interactions. Additionally, (Gr‐Gua‐C) has (−0.406239 eV), (Gr‐Gua‐N) has (−0.119104 eV), and (Gr‐Gua‐O) has (−0.447682 eV) also exhibit negative adsorption energies, denoting favorable interactions. The interaction with the most negative adsorption energy (−0.588474 eV) is the best interaction, indicating it is the most favorable interaction. Binding Distance (d): Shorter binding distances typically signify stronger interactions. The binding distances range from about 3.5723 A.U. to 6.1588 A.U. (Gr‐Cyt‐O) has a binding distance of 3.5723 A.U., one of the shortest distances, supporting its strong interaction as indicated by its adsorption energy.[^37^, ^38^, ^39^, ^40^]
- Q_Mu_ (Mulliken charge transfer)and Q_NBO_ (Natural Bond Orbital charge)‐ The Q_Mu_ and Q_NBO_ values indicate the transfer of electrical charge between atoms in a molecule, providing information about the direction and amount of electron transfer. A positive value indicates an atom gains electrons, while a negative value means losing electrons. The Q_Mu_ values range from −0.7860 e to 0.4070 e. Among these values, Gr‐Cyt‐N displays the most negative charge at −0.7860 e, showing a significant electron loss that could impact its interactions with other molecules. Likewise, Q_NBO_ values range from −0.7100 e to 0.6780 e, indicating the charge transfer direction. Negative values indicate electron loss, while positive values indicate electron gain.[^41^, ^42^]
- μD (Dipole Moment) ‐ values range from 2.0173 Debye to 9.2948 Debye. Higher dipole moments can indicate more polar interactions, potentially affecting adsorption.

The interaction with the best overall performance, (Gr‐Cyt‐O), has the most negative adsorption energy (−0.588474 eV) and relatively short binding distance (3.5723 A.U.).Additional observations reveal that other cytosine and guanine interactions also show favorable interactions but are not as strong as the Gr‐Cyt‐O interaction. Therefore, cytosine interacting through the oxygen site (Gr‐Cyt‐O) shows the strongest and most favorable interaction with the graphene surface based on the given data.[^37^, ^43^, ^44^]

### 3.3 HOMO, LUMO, and Energy Gap of DNA Nucleobases Adenine, Cytosine, and Guanine on Pure Graphene Surface

**Figure 4:**
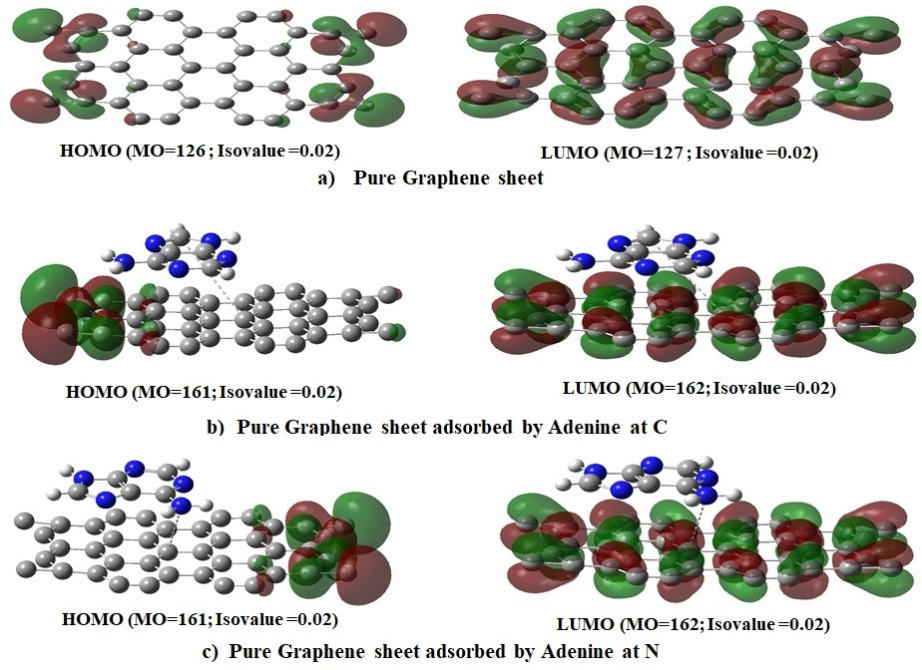
Scheme of HOMO‐LUMO levels of Gr &Gr‐Ade at different positions.

**Figure 5:**
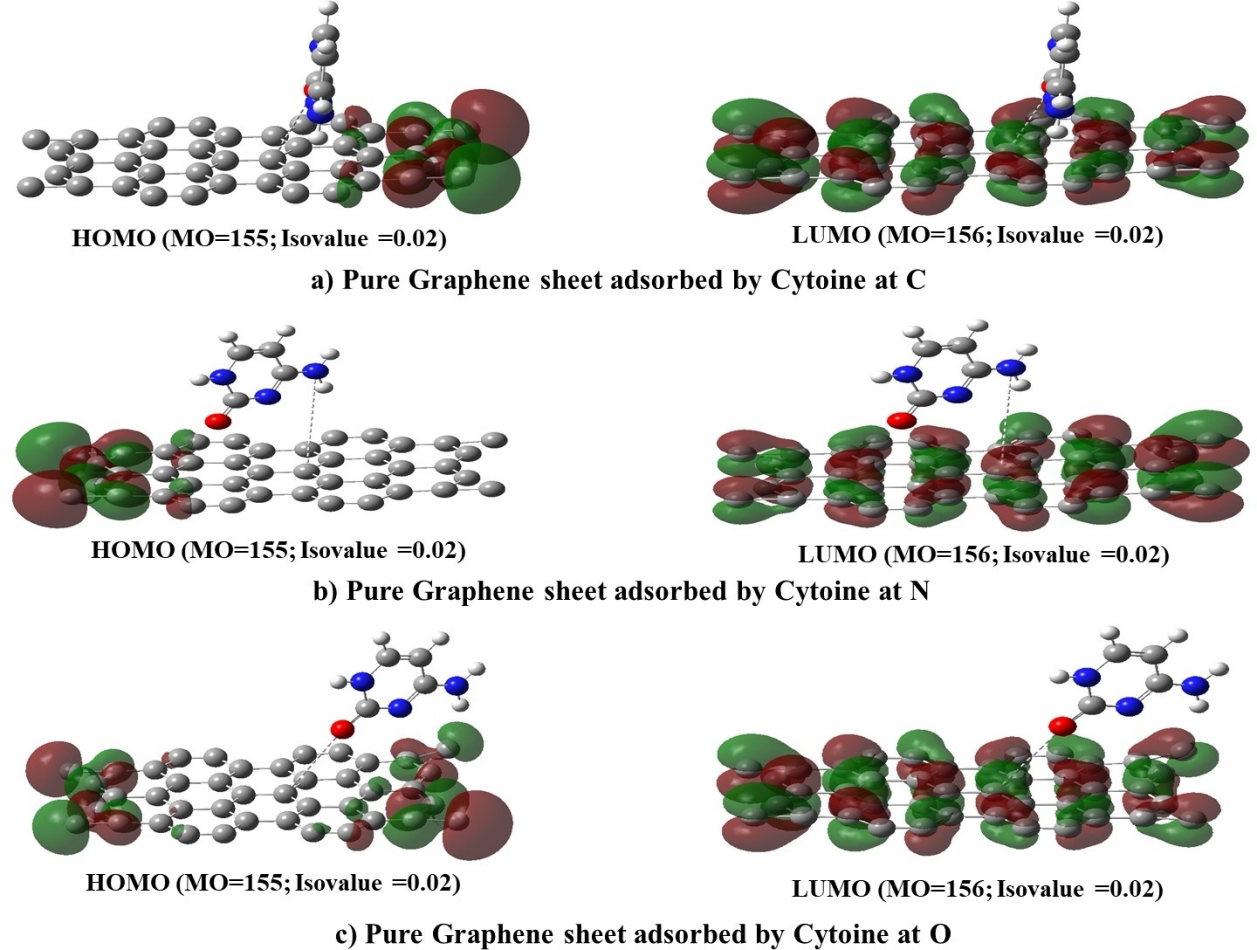
Scheme of HOMO‐LUMO levels of Gr‐Cyt at different positions.

**Figure 6:**
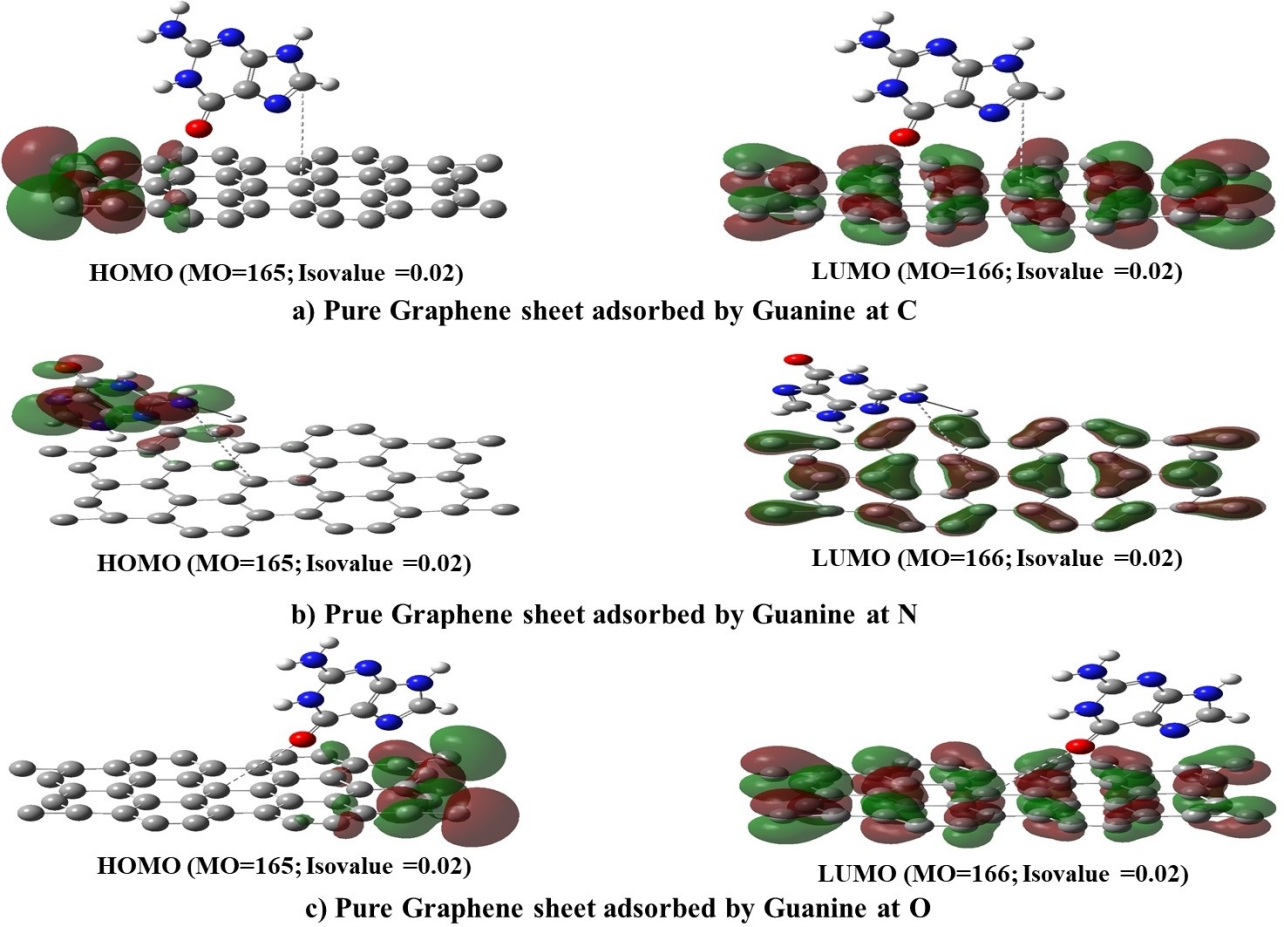
Scheme of HOMO‐LUMO levels of Gr‐Gua at different positions.

The calculation criteria were kept constant to enable a comparison of the properties of DNA nucleobases, including HOMO, LUMO, and energy gap. The resulting data for Pure Graphene with all DNA nucleobases is presented in Table 2 From the table the HOMO values range from −6.343 eV to −9.600 eV. Pure Graphene has a HOMO value of −6.775 eV, which is less negative compared to the Gr‐Ade systems. Gr‐Ade complexes have significantly lower (more negative) HOMO values, indicating more stability and less reactivity in these states. The LUMO values range from −4.487 eV to −2.846 eV. Pure Graphene has a LUMO value of −4.412 eV. The Gr‐Ade complexes have higher (less negative) LUMO values, making them more likely to accept electrons than others. The band gap energy ranges from 2.116 eV to 6.739 eV. Pure Graphene has a band gap of 2.363 eV. Gr‐Ade complexes have significantly larger band gaps (6.099 eV and 6.739 eV), suggesting they are less conductive and more insulating. Other complexes (Gr‐Cyt and Gr‐Gua) have band gaps close to that of Pure Graphene, indicating similar electronic properties. Gr‐Ade Complexes have the most negative HOMO values and the largest band gaps, indicating they are the most stable and least conductive among the complexes analyzed. Furthermore, Figures 4, 5, 6. provide visual representations of the obtained results.[^8^, ^20^, ^44^]

**Table 2:** HOMO‐LUMO and energy gap of complex biosystems Gr adsorbed by DNA nucleobases at at several locations.

Complex System	HOMO (ev)	LUMO (ev)	Eg (ev)
Pure Graphene	−6.775	−4.412	2.363
Gr‐Ade‐C	−8.945	−2.846	6.099
Gr‐Ade‐N	−9.600	−2.861	6.739
Gr‐Cyt‐C	−6.376	−3.994	2.382
Gr‐Cyt‐N	−6.384	−4.032	2.352
Gr‐Cyt‐O	−6.465	−3.991	2.474
Gr‐Gua‐C	−6.355	−3.955	2.400
Gr‐Gua‐N	−6.603	−4.487	2.116
Gr‐Gua‐O	−6.343	−3.952	2.391

### 3.4 Descriptors Global of Reactivity

- The analyze data on the electronegativity (χ), chemical hardness (η), chemical potential (μ), and electrophilicity (ω^2^) of graphene (Gr) adsorbed by DNA nucleobases at different positions according to Table 3
  **Table 3:** The Electronegativity (χ), Chemical Hardness (η), Chemical Potential (μ), And Electrophilicity (ω^2^) of complex biosystems Gr adsorbed by DNA nucleobases at different positions.

Complex System	χ (eV)	μ (eV)	η (eV)	ω^2^ (eV)
**Pure Graphene**	5.5932	−5.5932	1.1814	13.2402
**Gr‐Ade‐C**	5.8952	−5.8952	3.0494	5.6983
**Gr‐Ade‐N**	6.2307	−6.2307	3.3695	5.7609
**Gr‐Cyt‐C**	5.1854	−5.1854	1.1910	11.2878
**Gr‐Cyt‐N**	5.2081	−5.2081	1.1762	11.5305
**Gr‐Cyt‐O**	5.2276	−5.2276	1.2370	11.0457
**Gr‐Gua‐C**	5.1548	−5.1548	1.1999	11.0727
**Gr‐Gua‐N**	5.5454	−5.5454	1.0580	14.5332
**Gr‐Gua‐O**	5.1474	−5.1474	1.1955	11.0813
, we can break down the properties and compare how they change with different nucleobases and adsorption positions.
- Electronegativity (χ)‐ Increases significantly when graphene is adsorbed by adenine, especially at the nitrogen position. Slight changes when adsorbed by cytosine or guanine at different positions, with cytosine showing the lowest changes.
- Chemical Potential (μ)‐Follows the same trend as electronegativity but with negative values. Indicates a more negative potential for adenine, especially at the nitrogen position.
- Chemical Hardness (η)‐Highest for adenine, especially at the nitrogen position, indicating a high resistance to change in electron distribution. Lowest for guanine at the nitrogen position, suggesting more flexibility in electron distribution.
- Electrophilicity (ω^2^)‐Highest for Pure graphene and guanine at the nitrogen position, indicating strong electrophilicity. Lowest for adenine, suggesting that adsorption by adenine makes graphene less electrophilic.

Adenine adsorption significantly increases electronegativity and chemical potential, especially at the nitrogen position, while reducing electrophilicity. Cytosine and Guanine have more modest effects on graphene's properties, with guanine at the nitrogen position notably increasing electrophilicity. Pure graphene has high electrophilicity, which is altered by adsorption, with the degree of change depending on the specific nucleobase and adsorption site. The analysis highlights how different DNA nucleobases and their adsorption positions can significantly impact the electronic properties of graphene, which could have implications for its use in biosensors and other nanotechnological applications.[^43^, ^44^]

### 3.5 Density of States (DOS) of Complex Biosystems

**Figure 7:**
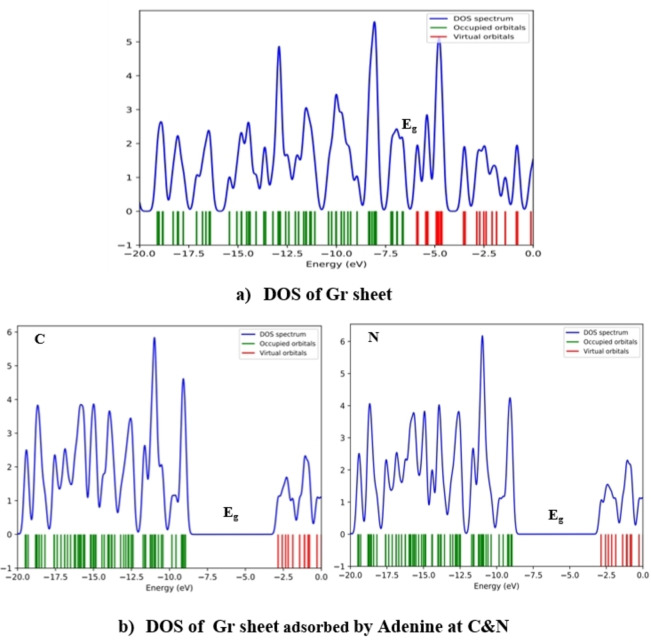
Scheme of DOS spectrum of Gr and Gr with Ade at different positions.

**Figure 8:**
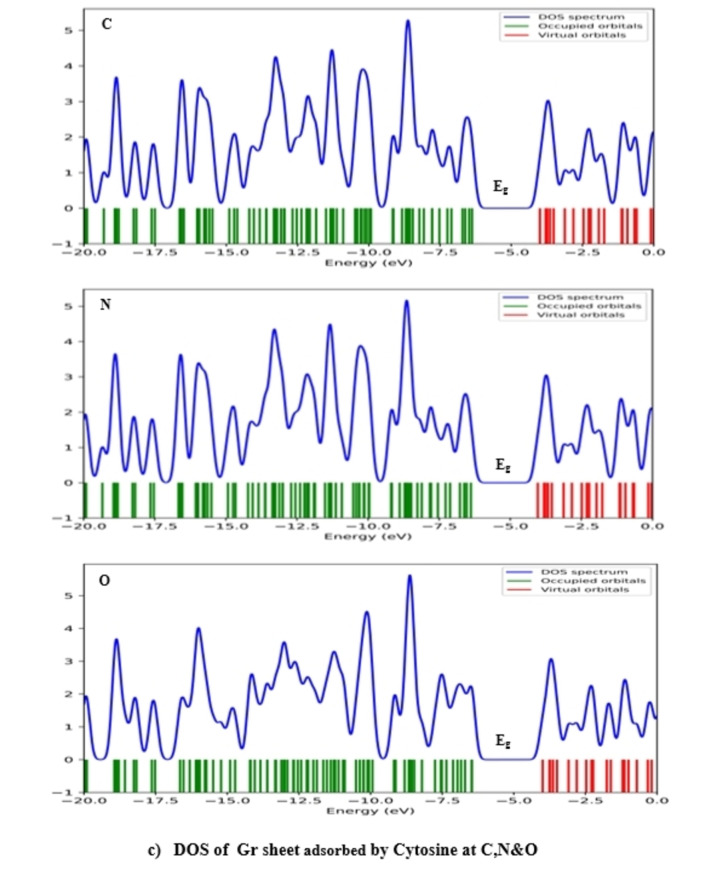
Scheme of DOS spectrum of Gr with Cyt at different positions.

**Figure 9:**
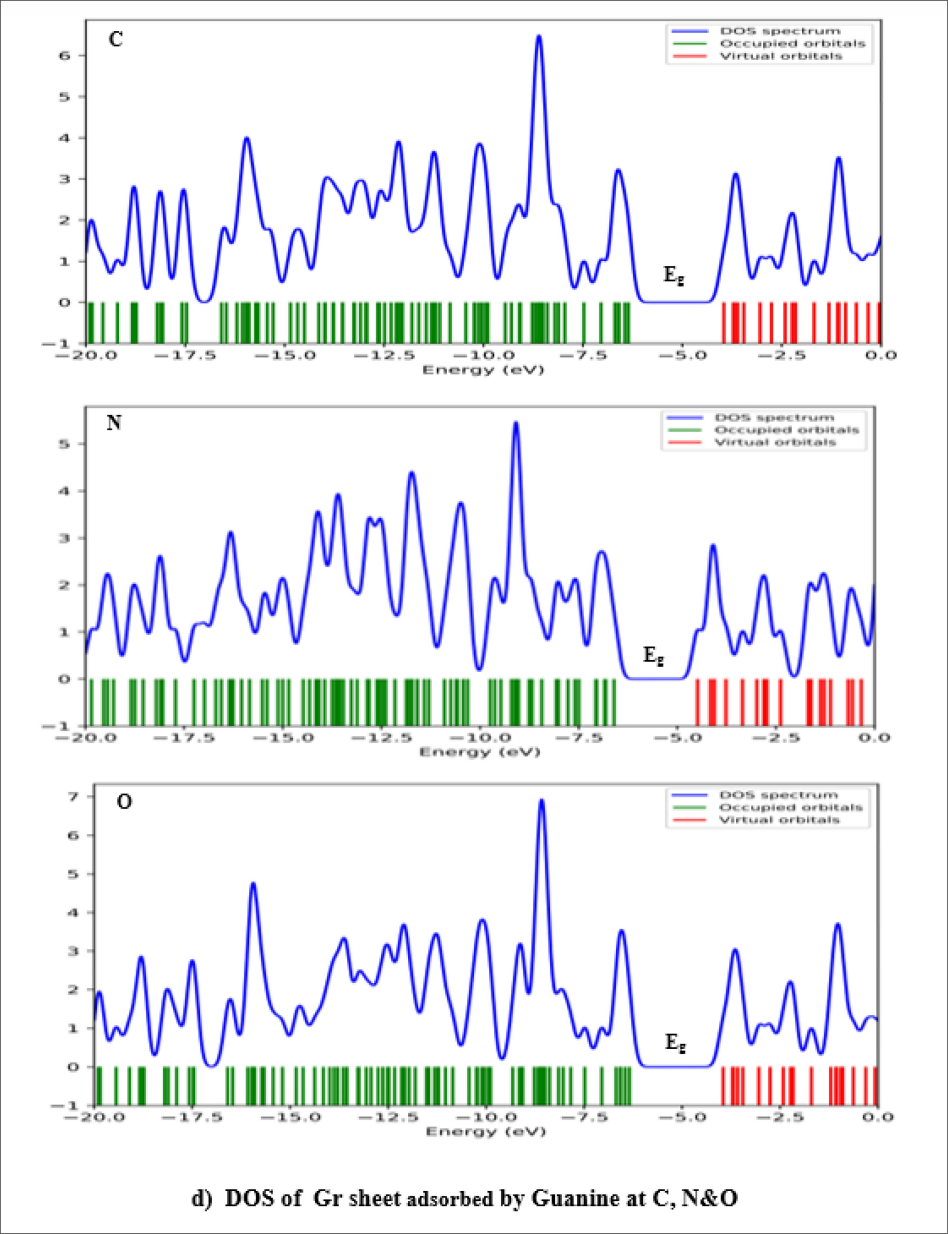
Scheme of DOS spectrum of Gr with Gua at different positions.

Figures 7, 8, 9 display the density of states, illustrating the distribution of electrons across different energy levels and showing the band bends. The valence band is green, and the conduction band is red. Local density of states results from system distortions, leading to localized vibrations around the equilibrium position. The electron density significantly influences the characteristics of the density of states. These states give us insight into the availability of energy levels within orbitals for electrons to occupy, thus revealing the quantum states where electrons can exist. The relationship between conductivity and E_g_ is given by Eq. 7:

(7) σαexp(Eg/kT)......

The variables in question are σ, which represents electrical conductivity, T, which represents temperature, and K, which represents the Boltzmann constant.

Equation demonstrates that as E_g_ value decreases, conductivity also increases. According to the extracted data in Table 2 Guanine has the smallest energy gap, which means it has the highest conductivity among the DNA nucleobases. Figures 7, 8, 9 effectively demonstrate the density of states in pure graphene and DNA nucleobases, providing valuable insights into their electronic properties and potential conductivity.[^19^, ^27^, ^45^]

## 4 Conclusions

We employed Density Functional Theory (DFT) to examine the capability of graphene‐based systems without any impurities to detect DNA nucleobases, specifically adenine, cytosine, and guanine. An analysis was conducted on several metrics, such as adsorption energy, HOMO‐LUMO energy levels, HOMO‐LUMO gap, dipole moment, charge transfer, and density of states, in order to gain insight into the structural and electrical features of these biosystems. The results indicate that the presence of guanine enhances the electrical conductivity of pure graphene by decreasing the HOMO‐LUMO gap energy. Moreover, graphene‐guanine complex demonstrates the utmost conductivity compared to other nucleobases that were examined. This quality positions it as a highly favourable option for the advancement of guanine‐based biosensors in the foreseeable future.

## Conflict of Interests

The authors declare no conflict of interest.

## Data Availability

The data that support the findings of this study are available from the corresponding author upon reasonable request.
